# Prevalence of Progressive Fibrosing Interstitial Lung Disease in Patients with Primary Sjogren Syndrome

**DOI:** 10.3390/jpm14070708

**Published:** 2024-07-01

**Authors:** Andreina Manfredi, Gianluca Sambataro, Alessandra Rai, Stefania Cerri, Domenico Sambataro, Caterina Vacchi, Giulia Cassone, Carlo Vancheri, Marco Sebastiani

**Affiliations:** 1 Rheumatology Unit, Azienda Ospedaliera Policlinico di Modena, 41100 Modena, Italy; 2 Department of Clinical and Experimental Medicine, Regional Referral Center for Rare Lung Disease, Policlinico “G. Rodolico-San Marco”, University of Catania, 95100 Catania, Italyvancheri@unict.it (C.V.); 3 Internal Medicine Unit, Department of Clinical and Experimental Medicine, Division of Rheumatology, Cannizzaro Hospital, University of Catania, 95100 Catania, Italy; 4 Artroreuma s.r.l., Rheumatology Outpatient Clinic, Mascalucia, 95030 Catania, Italy; 5 Rheumatology Unit, Azienda Unità Sanitaria Locale-IRCCS di Reggio Emilia, 42124 Medena, Italy; 6 Rheumatology Unit, University of Modena and Reggio Emilia, 42121 Reggio Emilia, Italy; 7 Respiratory Disease Unit, University of Modena and Reggio Emilia, 42121 Modena, Italy; 8 Rheumatology Unit, AUSL Piacenza, 42124 Piacenza, Italy; 9 Department of Medicine and Surgery, University of Parma, 43121 Parma, Italy

**Keywords:** primary Sjogren syndrome (pSS), interstitial lung disease (ILD), ILD-radiologic pattern, progressive pulmonary fibrosis (PPF)

## Abstract

Background: Interstitial lung disease (ILD) represents a frequent cause of morbidity and mortality in primary Sjogren syndrome (pSS). However, the prevalence and behavior of pSS-ILD remains incomplete, largely based on retrospective heterogeneous studies. Aim of the study: To investigate the prevalence of progressive pulmonary fibrosis (PPF) in a multicentric cohort of patients with pSS-ILD. Additionally, this study explored possible correlations between PPF and clinical, demographic, and serological features of pSS. Methods: All consecutive patients with pSS-ILD were enrolled in a 6-month period. Clinical, demographic, and serological features of pSS, other than functional and radiological lung features, were collected. Clinical behaviors of ILD other than PPF were also investigated. Results: Seventy-two patients were enrolled. A fibrosing ILD pattern was observed in 65.3% of patients with pSS-ILD; among them, 55.3% showed a PPF. The radiologic pattern (NSIP, UIP, or others) was not associated with PPF; in particular, patients with PFF had UIP in 42.3% of cases and NSIP in 57.7%, without a significant difference with respect to the non-PPF group (*p* = 0.29). Shorter pSS disease duration, higher age at pSS diagnosis, and lower frequency of antinuclear antibodies were correlated with the PPF subgroup. However, multivariate analysis did not confirm these associations. Discussion: This study provides valuable insights into the prevalence and characteristics of PPF in pSS-ILD. In particular, UIP and NSIP showed a similar evolution towards PPF in patients with pSS; for NSIP, this behavior was more frequent than for other rheumatic diseases. Our results emphasize the importance of early recognition of PPF for timely intervention and careful follow-up. Conclusions: This study provides valuable insights into the prevalence and characteristics of PPF in pSS-ILD. In particular, UIP and NSIP showed a similar evolution towards PPF in patients with pSS; for NSIP, this behavior was more frequent than for other rheumatic diseases. Our results emphasize the importance of early recognition of PPF for timely intervention and careful follow-up.

## 1. Introduction

Interstitial lung disease (ILD) stands as one of the most relevant pulmonary complications associated with primary Sjogren syndrome (pSS), leading to significant morbidity and mortality [1,2]. However, the available data on the prevalence and natural history are only partially known and mainly based on retrospective studies, resulting in considerable heterogeneity in ILD prevalence, ranging from 6% to 70% in various studies [3]. Recently, a systematic review and metanalysis, including 8255 patients with pSS in 30 studies, reported an estimated prevalence of ILD of 23% [3]. Similarly, the time of ILD onset in pSS is reported as highly variable, potentially affecting patients at any stage of the disease. Surprisingly, a percentage of patients between 10% and 51% develops ILD before the onset of the typical pSS symptoms, such as sicca syndrome [2].

PSS-related ILD may show the same radiologic patterns on high-resolution computed tomography (HRCT) observed in idiopathic interstitial pneumonias, such as nonspecific interstitial pneumonia (NSIP), usual interstitial pneumonia (UIP), organizing pneumonia (OP), and lymphocytic interstitial pneumonia (LIP) [3,4]. Among these patterns, NSIP is usually recognized as the most common in pSS, followed by OP and UIP [3,5,6]. Although Berardicurti et al., in the above reported metanalysis, described a frequency of 44% for UIP among pSS-ILD [3], the prevalence and clinical history of fibrosing patterns remain inadequately investigated in patients with pSS.

In particular, the early detection of progressive fibrosing patterns in pSS-ILD might be crucial since this lung complication generally leads to respiratory failure, in a variable period of time, in idiopathic interstitial pneumonia [7,8]. However, the latter is not frequently reported in the literature, and the prevalence of fibrosing patterns other than UIP remains inadequately investigated in pSS-ILD.

In fact, early detection of progressive fibrosing patterns in pSS-ILD might be crucial since this subgroup of lung complications, albeit over a variable period of time, generally leads to respiratory failure [7,8]. This disease course is well-known for idiopathic pulmonary fibrosis, which is characterized by a UIP pattern, but it is also described in fibrosing diseases secondary to rheumatic conditions, in particular rheumatoid arthritis (RA) and systemic sclerosis (SSc) [7,9,10].

Recently, a joint committee of the American Thoracic Society, European Respiratory Society, Japanese Respiratory Society, and Asociacion Latinoamericana de Torax (ATS/ERS/JRS/ALAT) provided a definition of progressive fibrosing ILD [11], suggesting the term progressive pulmonary fibrosis (PPS), despite treatment with nintedanib remaining limited to patients satisfying the inclusion criteria of the INBUILD trial [9]. The recent introduction of antifibrotic drugs has opened up the opportunity to treat fibrosing ILD in patients with pSS [9]. Although recent observations suggest that fibrosing patterns are quite common among subjects with pSS-ILD, data regarding the evolution over time of lung involvement in these patients is only partially available [12,13].

This study aimed to investigate the prevalence of progressive pulmonary fibrosis (PPF) among a cross-sectional cohort of unselected consecutive patients with pSS-ILD and to explore any possible correlations between PPF and the clinical, demographic, and serological features of pSS. The secondary aim of the study was to describe the clinical behavior of all other types of ILD.

## 2. Materials and Methods

We enrolled all consecutive patients with pSS-ILD who were referred to the multidisciplinary outpatient clinics (including pulmonologist and rheumatologist) of two Italian rheumatologic centers from July 2022 to January 2023. All patients fulfilled 2016 pSS classification criteria and had a diagnosis of ILD from at least 2 years before the enrolment [14]. ILD diagnosis was confirmed by chest high resolution computed tomography (HRCT). Moreover, all patients periodically underwent lung function tests (LFTs), including % of predicted forced vital capacity (FVC) and the % of predicted single-breath diffusing capacity of the lung for carbon monoxide (DLCO-SB) [15].

For each patient, demographic, clinical, and serological data, other than the more recent HRCT and LFTs, were recorded at the enrollment, while baseline FVC, DLCO, and HRCT scans were retrospectively collected.

According to INBUILD inclusion criteria, patients were defined as having a progressive ILD in cases of a relative decline in FVC ≥ 10% predicted and/or a relative decline in FVC ≥ 5% predicted, associated with an increased extent of fibrotic changes in chest imaging over a 24-month period [9]. Respiratory symptoms were excluded to reduce possible bias due to the retrospective interpretation of cough and dyspnea at baseline. A relative decline in FVC ≥ 10% predicted was considered also in patients with non-fibrotic lung disease to assess progression in this subgroup of subjects.

All participants provided written informed consent, and the present study has been approved by the local institutional ethics committee (approval number 108/2019).

Continuous variables were reported as mean and standard deviation (SD), while categorical variables were reported as absolute numbers and percentages. Categorical variables were analyzed via chi-square test or Fisher’s exact test when appropriate, and differences between the medians were determined using Mann–Whitney test for unpaired samples. Clinical features were reported as dichotomic or ordinal parameters. Then, a multivariate analysis was performed to analyze the effect of features at patient baseline regarding evolution to PPF. Analyses were made using the Statistic for Data Analysis software (IBM SPSS statistic, version 29, Armonk, NY, USA). A *p*-value < 0.05 was considered statistically significant [16].

### Radiologic Evaluation

HRCT was performed using different multidetector scanners with a slice thickness of less than 2 mm, from the lung apices to below the costophrenic angles, reconstructed using an edge-enhancing algorithm. The scan was performed in the supine position at full inspiration. All images were viewed at a window setting optimized for assessment of the lung parenchyma (width 1500 HU; level—700 HU). Chest HRCT scans were centrally assessed by an expert chest radiologist according to the Fleischner Society White Paper statement on the diagnosis of IPF [17]. The most recent HRCT, carried out within 3 months from the last available follow-up, was compared to HRCT performed 24 months before.

The HRCT pattern of disease was recorded as definite, probable usual interstitial pneumonia (UIP), or indeterminate for UIP. If a pattern indeterminate for UIP was noted, it was further classified as nonspecific interstitial pneumonia (NSIP), fibrotic NSIP, NSIP with organizing pneumonia (NSIP + OP), lymphocytic interstitial pneumonia (LIP), and other patterns [17,18].

Disease progression on HRCT was established in accordance with recently published guidelines on progressive pulmonary fibrosis (PPF) [11]. In particular, at follow-up, HRCT fibrotic disease progression was confirmed if one or more of the following changes were evident: increased extent or severity of traction bronchiectasis and bronchiectasis; new ground-glass opacity with traction bronchiectasis; new fine reticulation; increased extent or increased coarseness of reticular abnormality; new or increased honeycombing; and increased lobar volume loss [11].

## 3. Results

Seventy-two patients with pSS-ILD were enrolled in the study (males/females 14/58, median age 74 years [IQR 62–80], median p-SS duration 1 years [IQR 0–4], median ILD duration 2 years [IQR 1–3]). In 33.3% of cases, patients were current or past smokers; xerostomia and xeropthalmia were recorded in 66.7% and 69.4%, respectively. Antinuclear antibodies (ANA) were detected in 73.6% of patients; anti-SSA/Ro52 and anti-SSA/Ro60 were positive, respectively, in 27.8% and 40.3% of patients; among them, eight patients (11.1% of total population) were positive for both anti-SSA Ro52 and 60 kDa. More details about demographic, clinical, and serological data are reported in Table 1.

Diagnosis of ILD preceded that of pSS in 48.6% of cases, was concurrent in 26.4%, and followed pSS in 25% of patients.

With regard to radiologic patterns, ILD was classified as probable or definite UIP in 33.3% of patients, NSIP in 41.7%, NSIP + OP in 18.1%, and LIP in 2.8%; unclassifiable patterns were 4.2%. Considering the whole pSS-ILD population, ILD was classified as fibrosing in 47 (65.3%) patients (Table 2).

Treatment for pulmonary involvement was ongoing in 70.8% of patients with pSS-ILD, namely, azathioprine in 6 patients (8.3%), mycophenolate mofetil in 17 (23.6%), rituximab in 1 (1.4%), and nintedanib in 7 (9.7%). Globally, immunosuppressants were prescribed in 24 subjects (34.3%), in all cases except 3 in combination with a low dose of steroids. The dose of immunosuppressants remained unchanged during the period of the study, while nintedanib was added in seven progressive cases. Among these latter, all patients underwent low-dose corticosteroids (CS), in two cases in combination with immunosuppressants (azathioprine and mycophenolate mofetil, respectively). Oral CS were administered in 86.7% of patients, usually in a low dose (less of 10 mg of prednisone daily). Higher doses of CS were proposed only for short periods in cases of systemic exacerbation of pSS.

Median FVC at the time of the first evaluation was 91% (IQR 76–109), while DLCO was 59% (IQR 42–76). Globally, median value of FVC did not change during follow-up (median decline of 2%, IQR −10, +6), as well as DLCO (median improvement of 1%; IQR −8, +14). On the contrary, median FVC declined by 9.5% (IQR −15, −4) and DLCO by 1.5% (−20.75, +7) in the PPF population (Table 3).

### 3.1. Progressive Fibrosing ILD in pSS

Among the fibrosing patterns of ILD, a PPF was observed in 26/47 (55.3%) of cases (36.1% of the whole population). Patients with PFF presented a UIP pattern in 42.3% of cases and a fibrosing NSIP or NSIP + OP pattern in 57.7% of cases. Among patients with a fibrosing pattern of ILD, a relative decline of FVC ≥ 10% was observed in 9/47 subjects (19.1%) and a progression of the fibrotic features at HRCT with a relative decline of FVC ≥ 5% and <10% was observed in 8 (17%) subjects, while both displayed a relative decline of FVC ≥ 10% in other 9 (19.1%). As expected, median FVC and DLCO significantly declined in the PPF population over a 24-month follow-up period, with a decline of 9.5% (IQR −15, −4) and 1.5% (−20.75, +7) for FVC and DLCO, respectively.

To investigate features possibly correlated with PPF, we compared this subgroup with the rest of the population. At univariate analysis, a statistically significant difference was observed in regard to pSS disease duration, which was shorter in patients with PFF (median pSS disease duration: −13.5 vs. −1 months in PPF and non-PPF group, respectively; *p* = 0.011); age at pSS diagnosis, which resulted higher in patients with PPF-ILD (median age at pSS diagnosis: 73 vs. 65.5 years in PPF and non-PPF group, respectively; *p* = 0.013); and ANA positivity, less frequently observed in PPF (57.7% vs. 82.6% in PPF and non-PPF group, respectively; *p* = 0.028). These observations were not confirmed in multivariate analysis.

Interestingly, no difference was observed among PPF and non-PPF patients in regard to radiologic patterns; in particular, patients with PPF showed UIP in 42.3% and NSIP in the remaining 57.7%, without significant difference with the non-PPF group (*p* = 0.29).

### 3.2. pSS-ILD Other than PPF

Considering the whole population, 46 patients showed a pattern other than PPF; that is, 21/72 (29.2%) showed a fibrosing non-progressive pattern, 23/72 (31.9%) showed a non-fibrosing non-progressive pattern, while 2/72 (2.8%) showed a non-fibrosing progressive pattern. These latter two patients were one male and one female. Both subjects had positivity for rheumatoid factor, ANA, and anti-SSA and showed both eye and mouth dryness. A functional progression, with a reduction of FVC and DLCO, was recorded in both patients, but in one, a progression of the extension of ground-glass opacities was also observed. Mycophenolate mophetil was ongoing in one case, while CS (10–20 mg of prednisone daily) was prescribed in both patients.

## 4. Discussion

After the publication of the results of the INBUILD trial, a significant increased interest regarding ILDs other than IPF with a progressive fibrosing pattern was observed. This interest is encouraged by the potential for a targeted treatment approach for this specific patient population. It is widely accepted that fibrosing ILDs typically progress to respiratory failure, although the time and rate of progression can vary [6,8,11,19]. These insights originally derived from the data available for IPF, which, by definition, is characterized by a UIP pattern. Data from recent trials have clearly shown a similar clinical course for rheumatoid arthritis patients with UIP patterns also [20,21].

Characterization of pSS-ILD syndrome represents a hot topic that has been receiving increasing interest in recent years [22]. The radiologic findings of pSS-ILD were recently investigated in a systematic literature review, aiming to evaluate the prevalence and the most radiologic patterns of ILD in patients with pSS [3]. The authors analyzed 30 studies, including 8255 patients with pSS, and observed a pooled prevalence for ILD of 23% (95% CI: 16–30) among pSS subjects. The pooled prevalences of the different radiologic ILD patterns were 52% (CI 41–64) for NSIP and 44% (CI: 32–55) for UIP, respectively.

In a recent study, we investigated the prevalence of fibrosing ILD in patients with pSS, observing a high prevalence, higher than 50% of this pulmonary phenotype [12]. In any case, only a few studies aimed to investigate the clinical behavior of fibrosing ILD in patients with pSS.

To the best of our knowledge, this is one of the largest studies aiming to evaluate ILD progression in patients with pSS. In our study, ILD was detected before in conjunction with pSS diagnosis in the majority of cases, and NSIP pattern was observed in 59.9%, followed by UIP pattern in 33.3% of subjects.

Considering fibrosing and non-fibrosing patterns, 38.8% of patients showed a radiological or functional progression in a 24-month follow-up period.

In a recent study from Lee et al., the course and prognostic factors of 39 patients with pSS-ILD were retrospectively investigated, and a progressive behavior, considering both fibrosing and non-fibrosing patterns, was observed for 19 of them. The authors detected that in the progressive group of pSS-ILD, the extent of coarse reticulation and the coarseness score of fibrosis were significantly increased at follow-up, and that UIP pattern and follow-up duration were independent risk factors for disease progression in patients with pSS-ILD. Moreover, in both progressive and nonprogressive pSS-ILD, GGO decreased, whereas the extent of fibrosis increased even after treatment with glucocorticoid and/or immunosuppressants [23].

In our paper, we confirmed that a fibrosing pattern prevails in pSS-ILD, being detected in 65.3% of the whole population, and in 36.1% of the PPF population.

In recent years, an increasing number of researchers investigated the prevalence and the features of ILD in patients with pSS. As reported above, many authors determined that ILD could be an early manifestation of pSS, with a prevalence similar to that observed in other autoimmune systemic diseases, such as RA [24]. Moreover, the frequency of usual interstitial pneumonia and, in general, of fibrosing patterns is higher than that previously reported. Until now, only a few studies, with a small cohort of patients with pSS, evaluated the prevalence of PPF [25]; our study demonstrates a higher prevalence of PPF in pSS subjects also, very similar to that recently published in an Italian population of RA patients [26].

In 2021, a Korean study investigated the prevalence, risk factors, survival, and possible prognostic value of progressive fibrosing (PF)-ILD criteria in patients with fibrosing ILD other than idiopathic pulmonary fibrosis. Among patients with autoimmune diseases, 54 patients with pSS were enrolled together with RA-ILD and SSc-ILD patients. Among all patients with pSS-ILD, 21.4% showed a PPF [12,24,25]. Compared with this study, our results suggest a higher prevalence of PPF in pSS, similar to that observed for RA and SSc.

Recently, Chen et al. confirmed a high prevalence of PPF in pSS subjects. They investigated clinical outcomes and risk factors associated with progressive fibrosing interstitial lung disease (PF-ILD) in patients with pSS-ILD. Sixty-eight pSS-ILD cases were retrospectively identified. The prevalence of PF-ILD in patients with pSS-ILD was 50% (34/68), and the remaining 50% were considered to be stable or to have improvement, namely, they were the non-PF-ILD group. There were no significant differences in the demographics, pulmonary function tests, hemograms, autoimmune profiles, inflammatory markers, and HRCT patterns between PF-ILD and non-PF-ILD groups in terms of the baseline features [13].

Of interest, our patients with a PPF showed a UIP or a NSIP pattern at CT scan in 42.3% and 57.7% of cases, respectively, also considering patients with NSIP/OP features. These observations suggest, on the one hand, that the UIP pattern can also be frequently detected in pSS-ILD, showing a progressive behavior in almost the half of cases; on the other hand, it is interesting to observe that a similar evolution can be supposed for patients radiologically classified as exhibiting an NSIP pattern, more frequently than is observed for RA [26]. Our results for PPF prevalence are related to a partially arbitrary definition, one suggested in the enrolment criteria of the INBUILD trial and aimed to identify patients to be treated with antifibrotic drugs, namely, nintedanib [20]. However, in ILD related to rheumatic diseases, progression can also frequently occur in a longer period of time than 24 months [8]. For this reason, a careful evaluation of all patients with pSS-ILD should be assessed in clinical practice, with particular attention to early detection of fibrotic features.

Our study shows some limitations: first of all, the decision to avoid collecting data on dyspnea and cough might cause an underestimation of the prevalence of PPF, excluding symptomatic patients with a mild decline in lung function [8]; on the other hand, this choice improved the quality of data, limiting the collection exclusively to objective data. Other possible limitations of the study include the relatively small sample size and the absence of a comparison with patients with other types of ILD. Finally, our study has only partially investigated the potential effects of drugs on lung function decline in patients with PFF.

Some points remain to be defined. First of all, we evaluated our patients in a period of time spanning two years, according to the prescription criteria for nintedanib, but the time of progression may be quite different among ILD patients. Recently, Pugashetti demonstrated that prognosis of PPF is similar, regardless of the time of disease progression [8]. Moreover, in our study, the risk of progression is similar to that reported for other rheumatic disorders [24,25], but the predictive role of the HRCT ILD pattern seems to be different among such diseases.

Currently, we are not able to establish the risk/benefit ratio of an early treatment with anti-fibrotic therapy. Therefore, future studies should be aimed at investigating the predictive factors of ILD progression. Moreover, studies on specific rheumatic diseases should be addressed to identify possible differences in risk and time of ILD progression, prognosis, and management.

## 5. Conclusions

A close collaboration between rheumatologists and pulmonologists allows us to diagnose patients with pSS with lung-dominant disease early. These diagnoses are sometimes missing in rheumatologic cohorts, possibly inducing an underestimation of the prevalence of ILD in patients with pSS. A multidisciplinary evaluation also allows for better management of these patients, improving the therapeutic approach to pSS-ILD, particularly in patients with PPF. Only prospective studies could confirm our results and allow us to investigate the prognostic role of PPF in patients with pSS.

## Figures and Tables

**Table 1:** Demographic, clinical, and serological features of 72 patients with pSS-ILD.

Patients enrolled	72
Females/Males ^1^	14/58 (19.4/80.6)
Median age at pSS diagnosis ^2^	69.5 (60.25–75.75)
Median age at ILD diagnosis ^2^	67 (62–76)
Median age at enrollment ^2^	75 (62, 80)
Median interval pSS-ILD (months) ^2^	−6 (−28.25, 10.5)
Median pSS disease duration (years) ^2^	1 (0, 4)
Smoke ^1^	24 (33.3%)
Antinuclear antibodies ^1^	53 (73.6%)
Rheumatoid factor ^1^	20 (27.8%)
Anti-SSA (Ro60) ^1^	29 (40.3%)
Anti-SSA (Ro52) ^1^	20 (27.8%)
Anti-SSB ^1^	9 (12.5%)
Sicca Syndrome ^1^	65 (90.3%)
Eye dryness ^1^	50 (69.4%)
Mouth dryness ^1^	48 (66.7%)
Raynaud’s phenomenon ^1^	24 (33.3%)
Positive minor salivary gland biopsy ^1^	72 (100%)
Dyspnoea ^1^	57 (79.1%)
Dry cough ^1^	54 (75%)
FVC baseline ^2^	91 (76–109)
FVC 24 months ^2^	91 (76–110)
DLCO baseline ^2^	59 (42–76)
DLCO 24 months ^2^	64 (45–77)

^1^ Results are reported as number (percentage); ^2^ results are reported as median of percentage of predicted (interquartile range); unless specified data are referred at the enrolment; pSS-ILD: primary Sjogren’s syndrome-related interstitial lung disease; FVC: forced vital capacity; DLCO: diffusion lung of carbon monoxide.

**Table 2:** Radiologic patterns of pSS-ILD.

	%	*n*
Fibrosing	65.3	47
Non Fibrosing	34.7	25
LIP	2.8	2
NSIP	41.70	30
NSIP + OP	18.1	13
UIP	33.3	24
UIP indeterminate	4.2	3

pSS-ILD: primary Sjogren’s syndrome-related interstitial lung disease; LIP: lymphocytic interstitial pneumonia; NSIP: nonspecific interstitial pneumonia; OP: organizing pneumonia; UIP: usual interstitial pneumonia.

**Table 3:** Evolution over time of lung function in patients with pSS-ILD according to fibrosing pattern of lung disease.

		Fibrosing	Nonfibrosing	Fibrosing Progressive	Fibrosing Nonprogressive	Nonfibrosing Progressive	Nonfibrosing Nonprogressive
UIP ^1^		24 (100)	0	11 (45.8)	13 (54.2)	0	0
NSIP + NSIP/OP ^1^		23 (53.5)	20 (46.5)	15 (34.9)	8 (18.6)	0	20 (46.5)
Other ^1^		0	5 (100)	0	0	2 (40)	3 (60)
FVC baseline ^2^		89 (74–107)	93.5 (83–110.5)	86.5 (68.75–112.25)	99 (78.5–106.5)	99.5 (NA)	93 (68.75–112.25)
FVC 24 months ^2^		87 (74–109.25)	100.5 (82.5–113.75)	77.5 (63.25–96)	102.5 (83.75–111.5)	91 (NA)	100.5 (85–115.25)
DLCO baseline ^2^		56 (39–71)	62.5 (44.5–80.75)	54 (36.5–73)	60 (42–73)	64 (NA)	62.5 (45–80.25)
DLCO 24 Months ^2^		59 (44.25–72.5)	75 (64.5–80)	52 (42.5–66.25)	64 (53.25–79.75)	71 (NA)	75 (63–80.5)

^1^ Results are reported as number (percentage); ^2^ results are reported as median of percentage of predicted (interquartile range); pSS-ILD: primary Sjogren’s syndrome-related interstitial lung disease; LIP: lymphocytic interstitial pneumonia; NSIP: nonspecific interstitial pneumonia; OP: organizing pneumonia; UIP: usual interstitial pneumonia; FVC: forced vital capacity; DLCO: diffusion lung of carbon monoxide; NA: not applicable for the low number of patients.

## Data Availability

The data presented in this study are available on request from the corresponding author because they are still under analysis for other studies.
